# Non-invasive localization of post-infarct ventricular tachycardia exit sites to guide ablation planning: a computational deep learning platform utilizing the 12-lead electrocardiogram and intracardiac electrograms from implanted devices

**DOI:** 10.1093/europace/euac178

**Published:** 2022-11-12

**Authors:** Sofia Monaci, Shuang Qian, Karli Gillette, Esther Puyol-Antón, Rahul Mukherjee, Mark K Elliott, John Whitaker, Ronak Rajani, Mark O’Neill, Christopher A Rinaldi, Gernot Plank, Andrew P King, Martin J Bishop

**Affiliations:** Biomedical Engineering & Imaging Sciences, King’s College London, London SE1 7EH, United Kingdom; Biomedical Engineering & Imaging Sciences, King’s College London, London SE1 7EH, United Kingdom; Medical University of Graz, Graz 8036, Austria; Biomedical Engineering & Imaging Sciences, King’s College London, London SE1 7EH, United Kingdom; Biomedical Engineering & Imaging Sciences, King’s College London, London SE1 7EH, United Kingdom; Guy’s and St Thomas’ Hospital, London SE1 7EH, United Kingdom; Biomedical Engineering & Imaging Sciences, King’s College London, London SE1 7EH, United Kingdom; Guy’s and St Thomas’ Hospital, London SE1 7EH, United Kingdom; Biomedical Engineering & Imaging Sciences, King’s College London, London SE1 7EH, United Kingdom; Guy’s and St Thomas’ Hospital, London SE1 7EH, United Kingdom; Biomedical Engineering & Imaging Sciences, King’s College London, London SE1 7EH, United Kingdom; Guy’s and St Thomas’ Hospital, London SE1 7EH, United Kingdom; Biomedical Engineering & Imaging Sciences, King’s College London, London SE1 7EH, United Kingdom; Biomedical Engineering & Imaging Sciences, King’s College London, London SE1 7EH, United Kingdom; Guy’s and St Thomas’ Hospital, London SE1 7EH, United Kingdom; Medical University of Graz, Graz 8036, Austria; Biomedical Engineering & Imaging Sciences, King’s College London, London SE1 7EH, United Kingdom; Biomedical Engineering & Imaging Sciences, King’s College London, London SE1 7EH, United Kingdom

**Keywords:** Pre-procedure planning, Electrocardiogram, Implanted device electrograms, Non-invasive VT localization, Computational modelling, Deep learning

## Abstract

**Aims:**

Existing strategies that identify post-infarct ventricular tachycardia (VT) ablation target either employ invasive electrophysiological (EP) mapping or non-invasive modalities utilizing the electrocardiogram (ECG). Their success relies on localizing sites critical to the maintenance of the clinical arrhythmia, not always recorded on the 12-lead ECG. Targeting the clinical VT by utilizing electrograms (EGM) recordings stored in implanted devices may aid ablation planning, enhancing safety and speed and potentially reducing the need of VT induction. In this context, we aim to develop a non-invasive computational-deep learning (DL) platform to localize VT exit sites from surface ECGs and implanted device intracardiac EGMs.

**Methods and results:**

A library of ECGs and EGMs from simulated paced beats and representative post-infarct VTs was generated across five torso models. Traces were used to train DL algorithms to localize VT sites of earliest systolic activation; first tested on simulated data and then on a clinically induced VT to show applicability of our platform in clinical settings. Localization performance was estimated via localization errors (LEs) against known VT exit sites from simulations or clinical ablation targets. Surface ECGs successfully localized post-infarct VTs from simulated data with mean LE = 9.61 ± 2.61 mm across torsos. VT localization was successfully achieved from implanted device intracardiac EGMs with mean LE = 13.10 ± 2.36 mm. Finally, the clinically induced VT localization was in agreement with the clinical ablation volume.

**Conclusion:**

The proposed framework may be utilized for direct localization of post-infarct VTs from surface ECGs and/or implanted device EGMs, or in conjunction with efficient, patient-specific modelling, enhancing safety and speed of ablation planning.

**What’s new?**

- This is the first proof-of-concept study that successfully automates the localization of post-infarct VT from simulated implanted device EGMs (localization error < 15 mm) to target clinical VT episodes and reduce the need of VT induction.
- The localization performance from simulated ECG traces (<10 mm) outperforms existing clinical and algorithm-based approaches.
- We propose a computational-deep learning platform that could be of benefit to ablation planning when invasive mapping cannot be performed.

## Introduction

Catheter ablation (CA) is an established treatment option^1^ for the management of refractory, post-infarct ventricular tachycardia (VT). During the CA procedure, the localization of the anatomical sites—isthmus and/or VT exit site—critical to the initiation and maintenance of the VT^2^ is of primary importance in order to successfully reduce recurrence of future arrhythmic episodes. However, this task remains a significant clinical challenge, demonstrated by the approximate 50% recurrence rate within a year of VT ablation.^3^

Robust identification of optimal VT ablation targets necessitates invasive electrophysiological (EP) procedures with associated clinical risk despite use of state-of-the-art three-dimensional (3D) electroanatomic mapping (EAM) and other risk-reducing strategies. Although these strategies often allow accurate characterization of the clinical VT, this may be limited by non-inducibility or non-sustained and/or haemodynamically poorly tolerated VTs. Therefore, there exists a pressing need for effective non-invasive localization of VT circuits.

The surface 12-lead electrocardiogram (ECG) remains a critical tool^4^ in guiding pre-procedure planning for VT ablation. Non-invasive methods utilizing the ECG—such as ECG imaging (ECGi)^5^ and automated algorithms^6,7^—have been increasingly investigated to localize the VT. However, failure to record the ECG during the clinical arrhythmia limits their application, and there are concerns relating to its precision and accuracy compared to invasive modalities.

In recent studies, electrogram (EGM) recordings stored in implantable electronic devices have demonstrated their utility in guiding clinical^8,9^ and in-silico pace-mapping,^10^ helping characterize the clinical VT as well as facilitating automatic localization of focal VTs.^11^ Given that the majority of CA candidates already have a device *in situ*,^12^ the automation of post-infarct VT localization directly from EGM recordings could yield benefits to VT pre-procedure planning enhancing safety, speed and success of CA.

We set out to develop a non-invasive computational-deep learning (DL) platform to accurately localize critical VT exit sites from surface ECGs and intracardiac EGMs. We aimed to (i) automate post-infarct VT localization from the standard ECGs to improve on the performance of existing, ECG-based algorithms and (ii) carry-out a proof-of-concept study to assess the feasibility of utilizing implanted device EGMs in the preliminary investigation of post-infarct VTs, which may be of value to target clinical episodes, improving guidance of VT ablation, as well as potentially reducing the need of arrhythmia induction. In addition, we also sought to show the direct clinical application of our platform and computational investigation applied to VT localization from clinically recorded ECGs.

## Methods

### Overview of computational-DL framework

**Figure 1:**
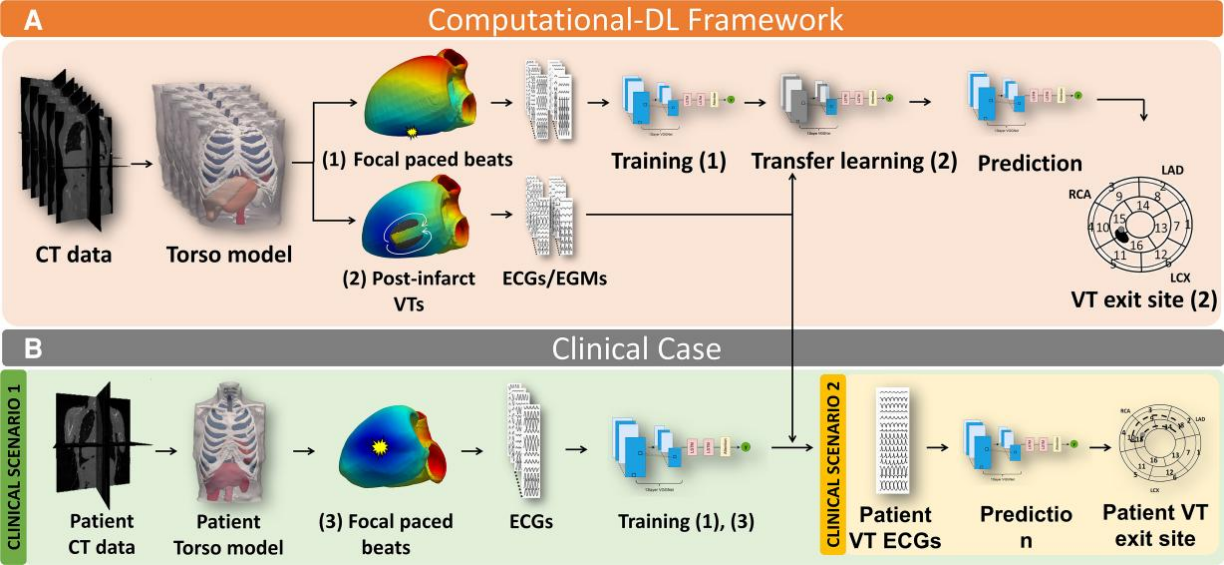
Workflow of this study. (*A*) Computational DL framework; (*B*) clinical applicability of the platform following clinical scenario 1 (green box) and clinical scenario 2 (yellow box). DL, deep learning.

Our framework to automate post-infarct VT localization is outlined in *Figure 1A*. Briefly, computational models from patient-specific anatomies were constructed and used to simulate multiple focal paced beats and episodes of post-infarct VT, along with the corresponding ECGs and implanted device EGM recordings. This comprehensive library of simulated time traces was then utilized to train a DL architecture to predict the exit sites of post-infarct VTs. The architecture was first evaluated on simulated post-infarct VTs and then tested on a clinically induced VT episode *Figure 1B*. The components of our pipeline are described in the following sections.

#### Patient-specific torso models

**Figure 2:**
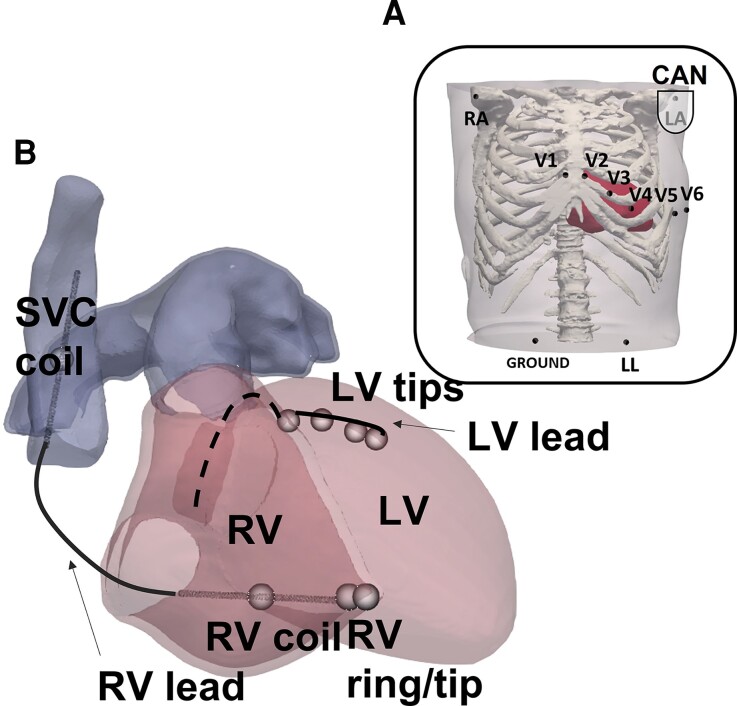
(*A*) ECG electrodes; (*B*) modelling of a generic implanted device. ECG, electrocardiogram.

CT imaging data from five patients were available for this study, including trans-catheter aortic valve implantation planning scans (whole torso), along with additional higher resolution contrast cardiac scans. All patients consented for the use of their data in ethically approved research: UK Research Ethics Committee reference number 19/HRA/0502 & 15/LO/1803. Torso models were constructed by segmenting major organs and tissues in Simpleware™ (Synopsys, Inc., Mountain View, USA) and cardiac chambers and blood pools in Siemens Axseg v4.11.^13^ 3D tetrahedral finite element meshes of all segmentations were then generated in Simpleware™ (see Supplementary material online, *Table S1* for mean ventricular edge lengths). Realistic myofiber architecture was incorporated using an established rule-based approach.^14^ In each torso, ECG electrodes were placed as per common clinical practice *Figure 2A*, and a generic implanted device was modelled as previously described.^10^ Briefly, as shown in *Figure 2B* and Supplementary material online, *Figure S1*, the device included a left-pectoral active can, an apical, dual-coil RV lead, and a quadripolar LV lead.

#### Simulation protocol

**Figure 3:**
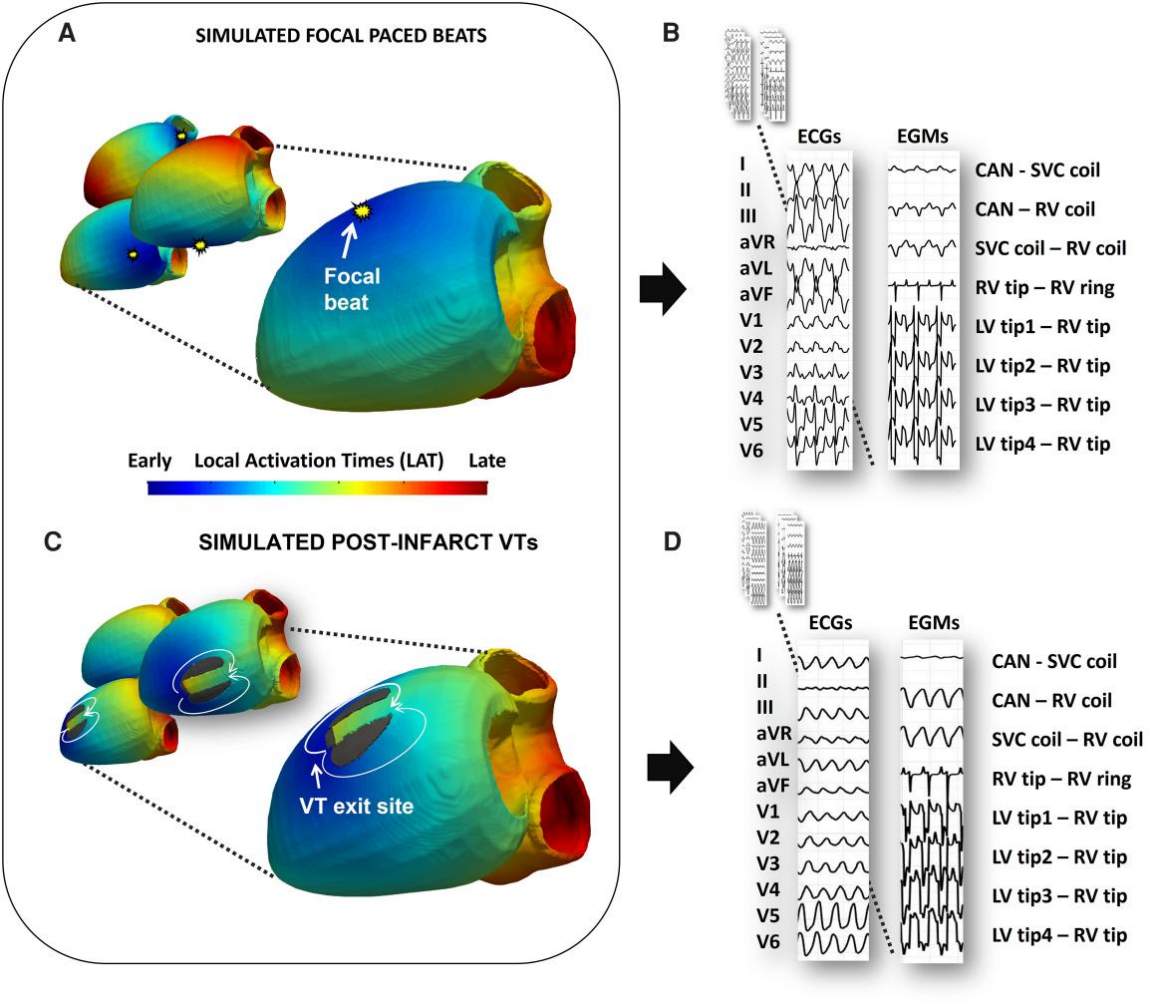
(*A*) Local activation time (LAT) maps of simulated focal paced beats; (*B*) corresponding ECGs and EGMs; (*C*) LAT maps of simulated post-infarct VT; (*D*): corresponding ECGs and EGMs. ECG, electrocardiogram; EGM, electrogram; VT, ventricular tachycardia.

Simulation of ∼3000 focal paced beats per model (*Figure 3A*) and corresponding 12-lead ECGs and 8-vector EGMs (*Figure 3B*) were generated using a computationally efficient reaction-eikonal (RE)—Lead-Field (LF)^15^ formulation within the Cardiac Arrhythmia Research Package (CARP),^16^ similar to our previous study.^11^

To rapidly simulate post-infarct VTs across the models, simplified, *virtual* infarcts were generated across the LV segments (see Supplementary material online, *Figure S2*). The scars were designed to produce the characteristic figure-of-eight re-entrant pattern, commonly associated with scar-related VT post-infarction,^17^ whereby two regions of inexcitable fibrosis frame a narrow region of reduced conduction (isthmus) (*Figure 3C*). Twelve-lead ECGs and eight-vector EGMs (*Figure 3D*) of these figure-of-eight post-infarct VT episodes were computed in a similar RE-LF simulation environment to above. See Supplementary Methods for further details.

#### DL model

**Figure 4:**
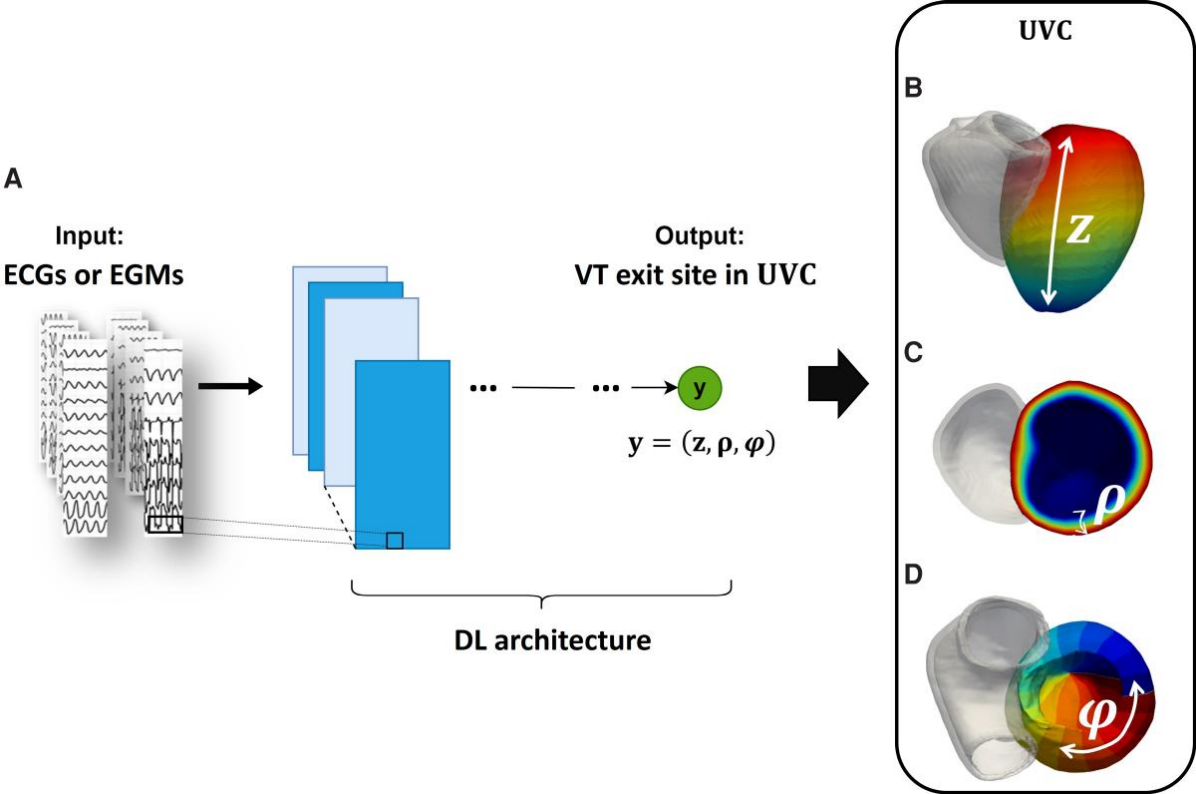
(*A*) ECG or EGM traces are the inputs to the DL architecture, which predicts VT exit site in universal ventricular coordinates shown in (*B*)—(*D*): (*z*, *ρ*, φ). DL, deep learning; ECG, electrocardiogram; EGM, electrogram; VT, ventricular tachycardia.

The DL model—described in more details in Supplementary Methods—takes either 12-lead ECGs, with the addition of 4 vector combinations as in previous studies,^11^ or 8-vector EGMs, as inputs (*Figure 4A*) and predicts VT exit sites, described below.

#### VT prediction

The DL model localizes the post-infarct VT exit sites in a standardized universal ventricular coordinate (UVC) system,^18^ defined by three parameters: *z*, apical-basal distance (*Figure 4B*); *ρ*, endocardial-epicardial distance (*Figure 4C*), and φ, rotational (circumferential) distance from the LV septum (*Figure 4D*). Performance of the model was expressed in terms of localization error (LE): Euclidean distance (in mm) between the model predictions and the known VT exit sites from simulations (the point of earliest systolic activation—see Supplementary Methods).

#### Training and testing

To be able to localize the exit sites of post-infarct VTs, the DL model was initially trained on the paced beat ECGs and EGMs. Then, part of the DL algorithm was re-trained on the simulated ECGs or EGMs of post-infarct VTs. This transfer of knowledge in the DL between two similar problems (paced beats and post-infarct VTs) is known as *Transfer Learning*. By initially training the networks on a large number of paced beats, which were less computationally expensive to simulate, we were able to reduce the number of post-infarct VT simulations required to achieve acceptable localization of the re-entrant episodes, while also providing important information to the networks during the initial pacing training (e.g. patient-specific anatomy, ECG/implanted device lead locations, relationship between extracellular potentials and locations in the heart). After training, we investigated two testing scenarios to predict simulated post-infarct VT exit sites: (i) we first tested the DL-framework on torso models that were *seen* during the initial pacing training, but *not* during VT transfer learning and (ii) we then tested how the DL would perform when a torso model was *unseen* during initial pacing training *and* VT transfer learning (see Supplementary Methods).

### Clinical case

CT and ECGi^19^ data from a patient with refractory VT were available to test our proposed computational-DL framework in clinical settings. The patient was recruited at Guy’s & St Thomas’ Hospitals NHS Foundation Trust, and later underwent successful cardiac stereotactive body radiotherapy (cSBRT), which was indicated due to previous extensive cardiac surgical interventions—mechanical aortic and mitral valve replacement precluding direct LV access for conventional VT ablation. The patient required an emergency bypass after the valve procedures, which suggested the presence of an ischaemic VT substrate. The clinical VT (cycle length ∼ 340ms) was induced with non-invasive programmed stimulation via the implanted cardiac resynchronization therapy defibrillator (CRT-D) device. The ablation target volumes—segment 9 and 10—were defined after careful clinical interpretation of the epicardial VT maps reconstructed from ECGi, and after measuring extracellular content volumes from CT to locate scar. Automated localization of the clinically-induced VT from ECGs—blinded to the clinical targets—was achieved following similar scenarios to the computational investigation described above.

#### *Clinical scenario 1*: VT prediction following computational simulations

A 3D torso model of the patient was constructed from patient CT data, in a similar manner to the torso models generated in **Patient-Specific Torso Models**. Only simple, paced beats were simulated on the newly generated myocardial mesh in ∼3000 randomly chosen locations, as previously described, along with corresponding ECGs. This data was used in combination with the paced beats of the previous five torso models, as additional training data, for the initial training of the DL algorithm. No post-infarct VTs were simulated on this patient’s mesh; transfer learning was used with the post-infarct VT data from the five previous torsos only. This methodology—no infarct modelling and simple paced beat simulations—was chosen to reduce computational load, while tailoring the DL training to the patient-specific anatomy and clinical settings. Finally, the clinically-induced VT exit site was predicted by the algorithm from the clinical ECG traces derived from ECGi (as described below).

#### *Clinical scenario 2*: direct VT prediction

Body surface potentials were collected with Medtronic CardioInsight Noninvasive 3D Mapping system,^19^ and utilized to compute standard 12-lead ECGs (with the additional four vectors as above) of the clinically induced VT. These were used as sole direct inputs to the previously trained DL architecture, returning the VT exit site with no prior knowledge on patient’s anatomy and EP modelling simulations.

## Results

### Evaluation of the computational-DL platform on simulated data

Firstly, we evaluated the localization performance of the proposed computational DL platform on simulated post-infarct VTs. Two scenarios were investigated. In *Scenario 1*, we localized *unseen* VT episodes from ECGs and EGMs of models *seen* during initial pacing training. In *Scenario 2*, we localized *unseen* VT episodes from ECGs and EGMs of models completely *unseen* during any training stage.

#### *Scenario 1*: VT prediction of models *seen* during pacing training

**Figure 5:**
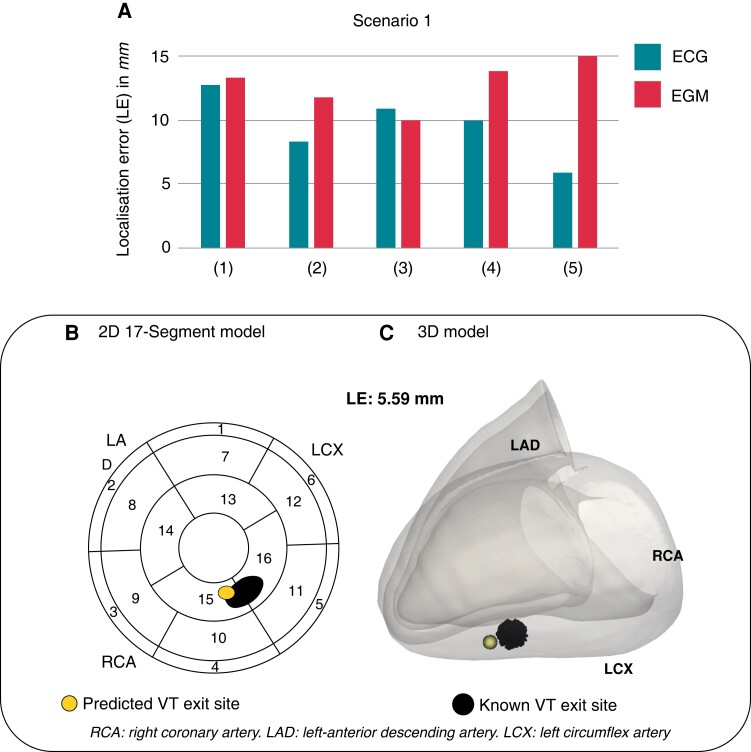
Computational scenario 1. (*A*) LEs across torso models 1–5. (*B*) and (*C*) Example of VT exit site prediction (yellow) and known VT exit site (black) in 2D and 3D. LE, localization error; VT, ventricular tachycardia.

We investigated the performance of our computational DL platform in localizing *unseen* simulated post-infarct VTs from ECGs and implanted device EGMs of models *seen* during pacing training. Data simulated on each torso model was sequentially excluded from transfer learning, and was utilized to test the algorithm in predicting post-infarct VT exit sites with limited knowledge on the torso model in question—only stemming from the initial training on focal paced beats. Across the torso models, the algorithm successfully localized post-infarct VTs from ECGs with mean LE 9.61 ± 2.61mm, ranging 5.92 − 12.76mm (*Figure 5A*). Encouragingly, the localization from intracardiac EGMs performed similarly to ECGs, with slightly higher mean LE across torsos 13.10 ± 2.36mm, ranging 10.04 − 16.36mm (*Figure 5A*). VT exit site predictions can be visualized in the standard 17-American Heart Association (AHA) LV model or in the patient-specific 3D myocardial mesh. An example of an ECG-based VT prediction for torso^2^ is shown *Figure 5B–C*, and is compared to the known VT exit site.

#### *Scenario 2*: VT predictions of models *unseen* during any training

**Figure 6:**
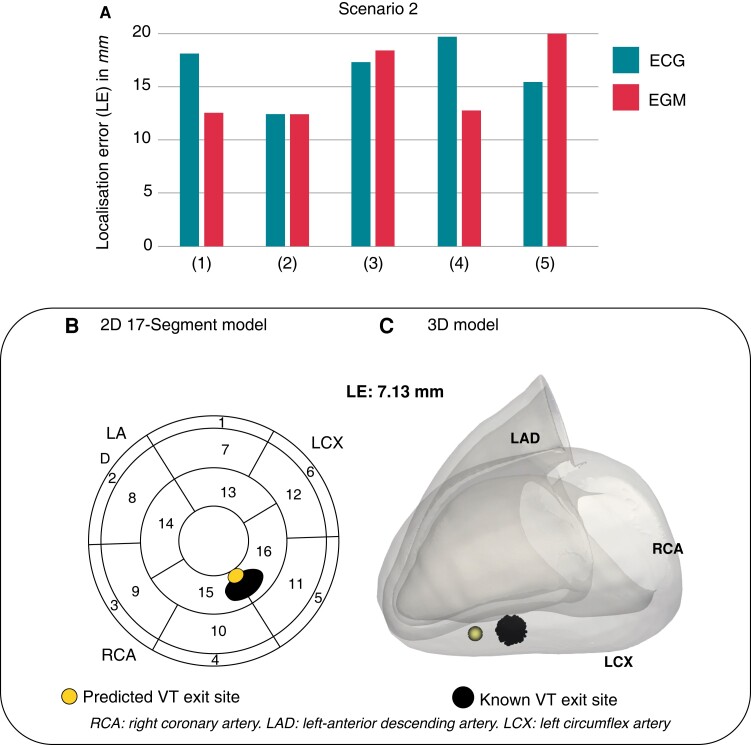
Computational scenario 2. (*A*) LEs across torso models 1–5. (*B*) and (*C*) Example of VT exit site prediction (yellow) and known VT exit site (black) in 2D and 3D. LE, localization error; VT, ventricular tachycardia.

We investigated how the absence of a patient-specific computational model during initial pacing training affected the localization performance of our DL algorithm in localizing entirely *unseen* simulated post-infarct VTs. In turn, data simulated on a torso model was excluded from both training stages; consequently, the DL model was tested on post-infarct VT signals with no prior knowledge of the torso in question. Across models, ECG-based localization returned mean LE 16.59 ± 2.79mm—ranging 12.41 − 19.68mm (*Figure 6A*). Successful localization of post-infarct VTs was also achieved utilizing EGMs, with a mean LE 15.80 ± 4.65mm, ranging 12.42 − 22.79mm (*Figure 6A*). Errors for ECG-based predictions increased in all five torsos compared to *Scenario 1* (Δ*LE* = 6.98mm), whereas EGM-based predictions seemed to be less affected by initial pacing training (Δ*LE* = 2.70mm). *Figure 6B–C* shows the ECG-based prediction of the same VT episode as in *Scenario 1*.

### Evaluation of the computational DL platform on clinical data

Finally, the localization performance of the proposed platform was evaluated on clinical data, following similar scenarios to the computational analysis. The DL architecture was tested on the patient’s VT ECGs *after* integrating imaging and simulation data into the pipeline (*Clinical Scenario 1*), and *directly* tested on the patient’s VT ECGs (*Clinical Scenario 2*).

#### *Clinical scenario 1*: VT prediction following computational simulations

**Figure 7:**
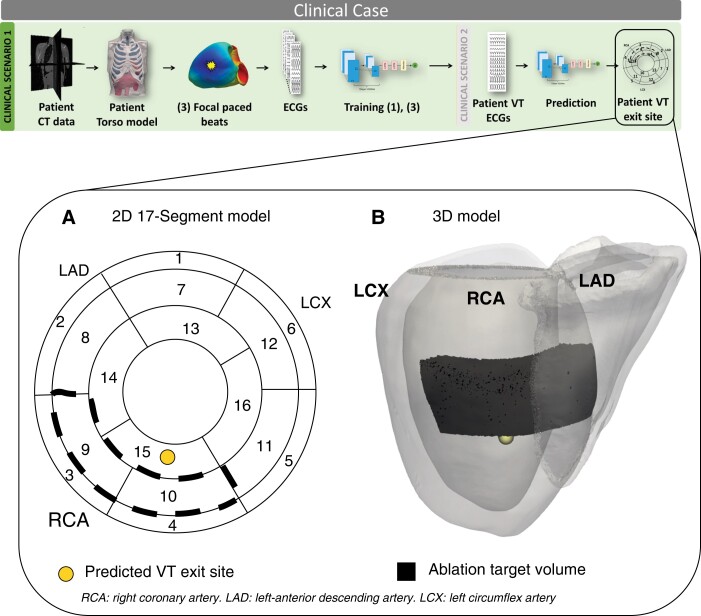
*Clinical scenario 1*: VT prediction following simulations. (*A*) and (*B*) VT exit site prediction (yellow) and ablation target volume (black) in 2D and 3D. VT, ventricular tachycardia.

We investigated the localization of the clinically induced VT episode after generating a patient-specific model from the patient imaging data, and utilizing the newly simulated data to train part of the DL model. With this increased patient-specific training data, the algorithm localized the VT episode in the upper part of segment 15, closer to both segment 9 and 10 (*Figure 7A–B*). Detailed (blinded) clinical analysis of epicardial activation patterns from the ECGi jacket data during the induced VT suggested VT exit site to be in segments 9 and 10.

#### *Clinical scenario 2*: direct VT prediction

**Figure 8:**
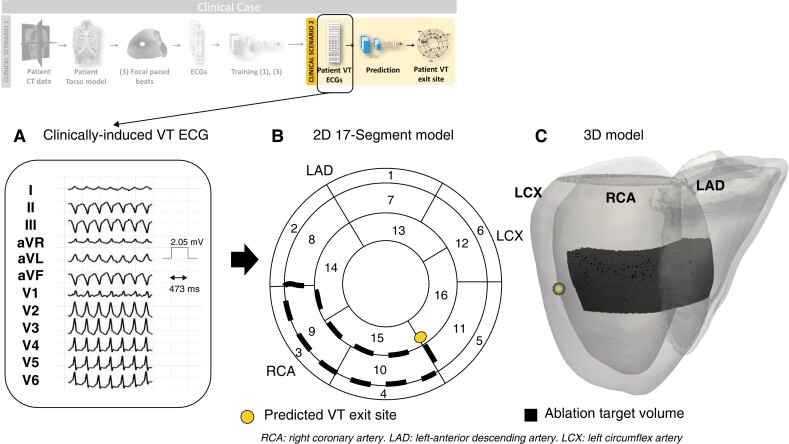
Clinical scenario 2: direct VT prediction. (*A*) Clinically induced VT ECGs, utilized as input to the trained DL model; (*B*) and (*C*) VT exit site prediction (yellow) and ablation target volume (black) in 2D and 3D. ECG, electrocardiogram; VT, ventricular tachycardia.

The DL model trained on simulated data of all five torsos was directly tested on the ECG traces (*Figure 8A*) recorded during the clinically-induced VT. The model prediction was now located in the inferior-apical part of segment 11, in close proximity to segment 10 (*Figure 8B–C*).

## Discussion

In this study, we proposed a novel, non-invasive platform for automating the localization of post-infarct VTs from extracellular potential signals to guide pre-procedural planning. We (i) demonstrated that ECGs can be utilized to accurately and reliably localize post-infarct VT exit sites, (ii) showed the feasibility of utilizing stored intracardiac EGM recordings from implantable devices to localize VT exit sites with comparable precision to surface ECGs, that would be acceptable for pre-procedure planning and (iii) demonstrated the applicability of our platform and computational investigation in clinical settings.

### Successful localization of post-infarct VTs from surface ECGs

We successfully localized post-infarct VT exit sites from ECGs with errors 9.61 ± 2.61mm and 16.59 ± 2.79mm in *Scenario 1* (VT prediction of models *seen* during pacing training), and *Scenario 2* (VT predictions of models *unseen* during pacing training and VT transfer learning), respectively. Ablation of post-infarct VTs often requires extensive lesions. Hence, LE<= 17mm may be deemed clinically useful in identifying initial VT ablation targets and guiding pre-planning procedures non-invasively and efficiently, and represents improvement over previous methods which localized only to the nearest AHA segment.^7^

Our developed DL-platform was seen to be robust to differences in patient anatomies, with little variation in LEs witnessed across torso models (standard deviation < 3mm in both *Scenario 1* and *2*). However, lower mean LEs in *Scenario 1* compared to *Scenario 2* showed the higher accuracy of the network in localizing post-infarct VTs when a computational model of the patient was created and *seen* during pacing training. Such behaviour of the DL-platform is expected, as the more patient-specific data that is provided to the network (*Scenario 1*), the better the latter performs when applied to that patient. Nevertheless, the localization performance in *Scenario 2* may be of benefit for immediate identification of VT critical sites (requiring *only* patient ECG traces as input), thus showing the utility of our platform in absence of imaging data.

#### Clinical implications

The majority of current methods that utilize the ECG to predict VT substrates are either qualitative^4,6^ or algorithm-based,^7^ trained on clinical VT ECGs. The former is extremely useful for preliminary assessment of idiopathic VT, but fails at robustly localizing more complex post-infarct VTs, and relies on clinical expertise. Algorithm-based methods can automatically localize the VT, but only within different LV anatomical regions with areas >10cm^2^.^20^ Moreover, they do not consider patient-specific geometries, and are trained on limited VT ECG libraries. Other automated methods that are trained on larger datasets and/or computational simulations can only locate premature ventricular contractions driven by focal VTs.^11^ Recently, ECGi^5^ has showed promise in characterizing VTs non-invasively, by reconstructing epicardial unipolar electrograms using body surface potentials. ECGi can be utilized to localize epicardial VT sources (with average resolution ∼ 13.2mm^5^), but fails at identifying septal, intramural and/or endocardial substrates. To the best of our knowledge, this is the first study that successfully automates the localization of post-infarct re-entrant VTs, achieving a 3D localization precision that outperforms existing ECG-based modalities.

### Successful localization of post-infarct VTs from intracardiac EGMs

We successfully localized post-infarct VT exit sites from generic implanted device EGMs with overall LEs < 16*mm*, comparable to ECG. Little dependence on anatomical differences between the in-silico patients was seen, similar to ECG-based localization; however, a smaller increase in LEs was witnessed between the different scenarios (Δ*LE* = 2.70mm) compared to ECG-based localization (Δ*LE* = 6.98mm). This finding shows the reliability of stored EGM recordings for direct localization of the clinical VT, when the latter is non-inducible or unmappable, and shows great potential for future applications to guide non-invasive ablation therapies, such as cSBRT.

#### Clinical implications

The majority of existing invasive, and non-invasive, mapping modalities rely on identifying and targeting an induced clinical arrhythmia, which is often not possible and/or not hemodynamically tolerable. Recent clinical studies^8,9^ demonstrated the feasibility and utility of implantable cardioverter defibrillator (ICD) EGMs in targeting the clinical VT during pace-mapping. Although a lower incidence in VT recurrence was found^9^ when targeting non-inducible clinical VTs from ICD EGMs, higher errors in localizing VT exit sites were reported^8^ compared to ECG-based maps. In our previous in-silico pace-mapping study^10^ we demonstrated how EGM-based pace-mapping resolution may be enhanced by utilizing signals from devices with both dual-coil RV and multipolar LV leads by increasing the number of sensing vectors used. We subsequently showed the utility of multiple sensing EGM vectors to automate focal VT localization in a similar computational-DL framework^11^ as presented here. In this present work, we go further to show the enhanced information contained in sensed EGMs from multi-polar devices helps achieve robust performance in the localization of post-infarct VTs.

### Applicability of our platform in clinical settings

We showed the applicability of our computational DL platform to localize a clinical VT episode. In both scenarios investigated, we localized the VT exit in proximity to segment 10, which was consistent with the clinical ablation targets (segments 9 and 10). According to an initial clinical investigation of the patient, the VT exit site was located in segment 10, with possible scarred tissue present in segment 9 (derived from ECVs). It is important to mention that while *Clinical Scenario 2*—direct VT prediction from ECGs *without* the creation of a patient-specific computational model—can return immediate results, with acceptable accuracy, the creation of a patient-specific model and integration of simple pacing simulations into the pipeline (*Clinical Scenario 1*) can increase localization accuracy further, at a low relatively computational expense. Thus, in patients for which invasive mapping cannot be performed, our computational-DL platform may provide rapid ECG-based localization of post-infarct VTs, accompanied by a more thorough patient-specific analysis, if CT imaging data are available. In future studies, this work might be of importance to investigate alternative, non-invasive tools to aid ablation procedures (e.g. cSBRT) where invasive mapping cannot be performed. Although implanted device EGMs of this patient were not available to evaluate EGM-based localization performance, from our computational analysis carried-out in the previous sections, we believe that EGM-based localization of the clinical VT would be similar to ECG-based.

### Study limitations

Our library of post-infarct VTs was limited to relatively simplistic figure-of-eight VT circuits. Despite providing robust training data that facilitated localization of acceptable accuracy, in the future, it would be useful to include more complex infarct geometries, integrated from multi-modal imaging with latest image-analysis software for instance, and VTs in the training, to investigate their impact in the final localization. This may help refine the training of our DL framework further, and may improve the results of our predictions. However, ideally our proposed approach would be used with minimal computational model construction and simulation for a particular patient (*Clinical Scenario 2*). Our clinical validation was limited to one patient, due to limited availability of clinical data, for which EGM data could not be retrieved. In clinical practice, retrieving eight-lead EGMs may be challenging from the current generation of implanted cardiac devices, as many implanted patients do not have an SVC coil, or multipolar LV lead, and/or routinely store only two EGM vectors simultaneously. Nonetheless, device technology and design are constantly evolving, which may open-up possibilities of additional EGM sensing vectors, driving VT management beyond using the standard ECG. In this context, our work is of importance to demonstrate the potential of implanted devices, and the necessity of improving their programming, design, and remote monitoring capabilities. In this present work, we chose to make use of cSBRT patient data as both high quality CT imaging data and non-invasive VT induction and mapping via ECGi were available, which enabled full torso model construction and comparison of our prediction with the clinically-induced VT exit site. In our institution, this type of data was not available for conventional VT ablation patients, for which substrate-based ablation is routinely performed (where the VT site of origin/exit site is therefore not localized). However, we hope to address these limitations in the future, and expand the clinical evaluation of our platform to a larger cohort of CA patients in order to quantify localization performance against more specific ablation targets.

## Conclusions

Our proposed computational DL framework may be utilized for direct localization of post-infarct VT exit sites from ECGs and/or implanted device EGMs, in absence of patient imaging data, or in combination with computationally efficient, patient-specific modelling, ultimately enhancing safety and speed of pre-procedure ablation planning.

## Supplementary Material

euac178_Supplementary_Data

## Data Availability

Fully anonymized imaging data will be shared where patient consent allows, on reasonable request to the corresponding author, and so torso models, simulated ECGs/EGMs, and trained DL models.
