# Supplementary material for: Non-invasive localization of post-infarct ventricular tachycardia exit sites to guide ablation planning: a computational deep learning platform utilizing the 12-lead electrocardiogram and intracardiac electrograms from implanted devices
Source: Europace. 2022 Nov 12;25(2):469–77. doi: 10.1093/europace/euac178 (PMC9935046; doi:10.1093/europace/euac178)
Supplement: euac178_Supplementary_Data [file euac178_supplementary_data.docx]

# **Supplementary Methods**

*Non-invasive Localisation of Post-Infarct Ventricular Tachycardia Exit sites to Guide Ablation Planning: A Computational-Deep Learning Platform Utilising the 12 -lead Electrocardiogram and Intracardiac Electrograms from Implanted Devices*

Sofia Monaci, Shuang Qian, Karli Gillette, Esther Puyol-Antón, Rahul Mukherjee, Mark K. Elliott, John Whitaker, Ronak Rajani, Mark O’Neill, Christopher A. Rinaldi, Gernot Plank, Andrew P. King, Martin J. Bishop.

## **Patient-specific torso models**

The resolutions of the five CT TAVI planning scans, and corresponding contrast cardiac scans, are reported in **Table S1**. Each CT TAVI planning scan was segmented into the major organs and tissues - lungs, bones, skin, liver, spleen, kidneys, stomach, aorta, fat and muscle. Each cardiac scan was segmented into separate chambers and blood pools. The five patients did not present any visible structural heart disease. Mean edge length in each myocardial mesh is reported in **Table S1**. **Figure S1** illustrates how we modelled the standard 9 ECG electrodes and generic implanted devices in all five torsos, following previous studies(1,2). All virtual devices had a non-septal RV lead(3) (with a sensing coil in the RV blood pool, and a sensing ring and tip touching RV apex), with a superior vena cava (SVC) coil in the right atrium (RA), and a straight LV epicardial lead with four sensing tips(4) distanced equally at $7.5 mm$. We approximated all sensing coils and tips to single points.

## **EP properties**

EP properties were assigned to each patient-specific model according to literature, given that no personalised EP data was available. Extracellular tissue conductivities of thoracic and abdominal organs and tissues are reported in **Table S2**(5). Intra- and extra-cellular conductivities of the myocardial meshes were tuned according to mesh resolution to achieve physiological QRSs(6). In all five models, the conduction velocities along and transverse to fiber direction were $0.67 m/s$ and $0.30 m/s$, respectively. The Ten Tusscher ventricular cell model(7) was utilised in all our simulation protocols, describing with high fidelity human ventricular action potential (AP).

| **Patient** | **Resolution CT TAVI planning scan** | **Resolution CT cardiac scan** | **Ventricular Mesh Resolution** |
| --- | --- | --- | --- |
| (A) | $0.6875 x 0.6875 x 0.5 mm$ | $0.4082 x 0.4082 x 0.5 mm$ | $835 \mu m$ |
| (B) | $0.6914 x 0.6914 x 0.5 mm$ | $0.3164 x 0.3164 x 0.5 mm$ | $765 \mu m$ |
| (C) | $0.6914 x 0.6914 x 0.5 mm$ | $0.3027 x 0.3027 x 0.5 mm$ | $778 \mu m$ |
| (D) | $0.7421 x 0.7421 x 0.5 mm$ | $0.4004 x 0.4004 x 0.5 mm$ | $826 \mu m$ |
| (E) | $0.6542 x 0.6542 x 0.5 mm$ | $0.3613 x 0.3613 x 0.5 mm$ | $826 \mu m$ |

Table S1. Resolutions of CT TAVI planning, cardiac scans and ventricular meshes (left to right) for five anonymised patients - (A), (B), (C), (D) and (E).


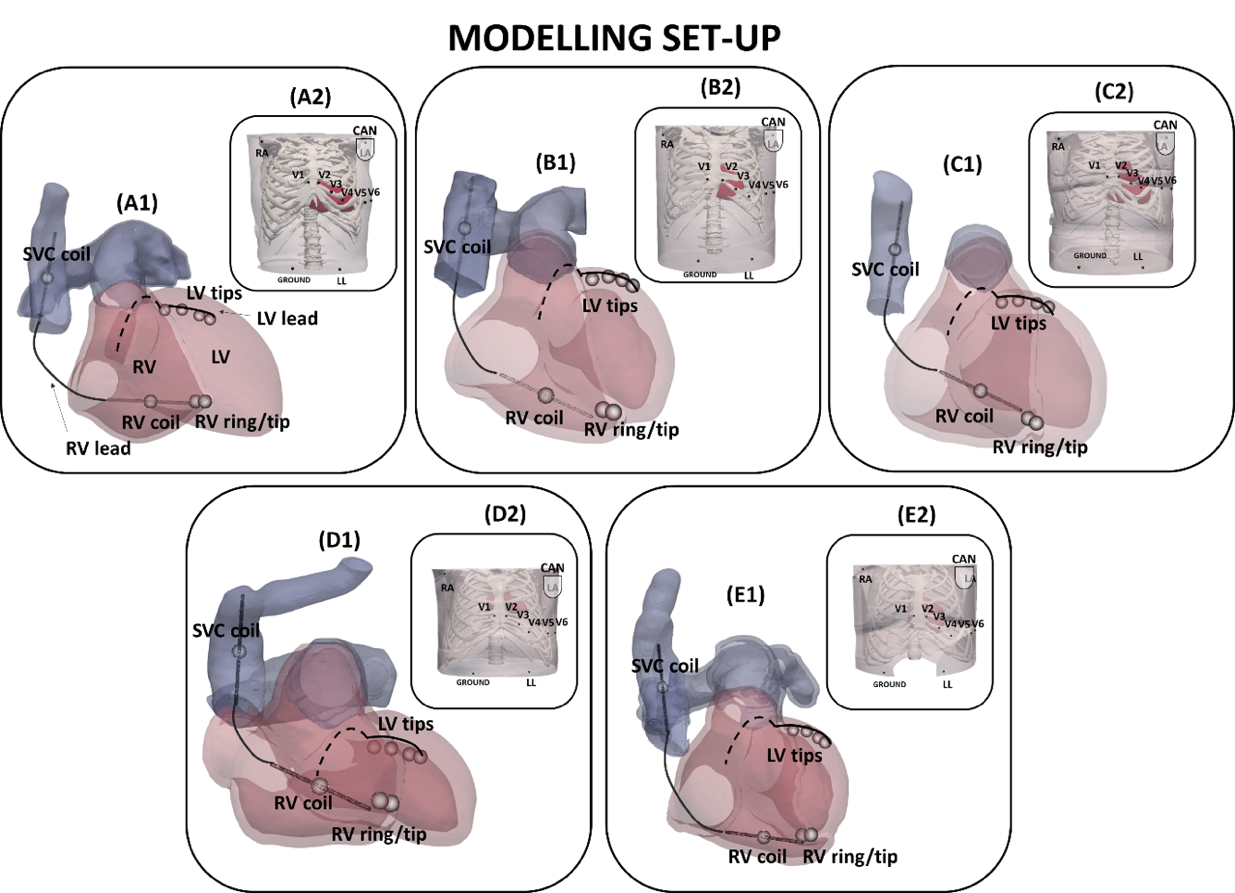


Figure S1. Modelling of implanted device leads (1) and ECG electrodes (2) in patient-specific models (A) – (E).

| **Organs** | **Tissue conductivities (S/m)** |
| --- | --- |
| Lungs | 0.0714 |
| Bones | 0.05 |
| Skin | 0.05 |
| Fat/Muscle | 0.24275 |
| Liver | 0.1667 |
| Spleen | 0.1 |
| Kidneys | 0.1667 |
| Stomach | 0.1 |
| Aorta, blood pools | 0.6667 |

Table S2. Tissue conductivities (S/m) assigned to major organs and tissues.

## **RE-LF environment**

Reaction-Eikonal (RE) formulation(8) allows to solve cardiac electrical propagation on a mesh with coarse spatial resolution; local activation times (LAT) – computed with the eikonal model – mediate the propagation of the electrical wave, returning transmembrane potential distributions at a reduced computational cost compared to reaction-diffusion models (where the wavefront propagation is mediated *by diffusion*). The Lead-Field LF method(9,10) consists of computing and storing extracellular potentials on a limited number of points. By combining RE with LF, we were therefore able to compute extracellular potential signals (e.g. ECGs and EGMs) on specific torso locations for different propagation patters, significantly reducing simulation time compared to conventional bidomain(11) and/or pseudo-domain(12) formulations. In this study, for each torso model, LF matrices were computed on the standard 9 ECG electrodes and on the approximated implanted device leads. The signals generated for different simulation protocols were the standard 12-lead ECGs, with the addition of 4 vector combinations (LL-RA-LA, LA-RA-LL, RA-LA-LL, (RA+LA+LL)/2)(2) – and 8 EGM vector combinations from the implanted devices: far-field CAN – SVC coil, CAN – RV coil, and SVC coil – RV coil, and near-field RV tip – RV ring, and each LV tip – RV tip.

## **Simulation protocol: focal paced beats**

For each model, multiple focal paced beats were simulated in the previously described RE-LF environment by pacing ~ 3000 randomly chosen locations (endocardially, epicardially and midmyocardially) across the LV American-Heart-Association(13) segments. Each location was stimulated twice ($bcl = 400ms$).

## **Simulation protocol: post-infarct VTs**

Idealised transmural infarcts were generated in 50 different locations in each torso model, for a total of 250 scars. The direction of the isthmuses changed according to fiber orientation(14) – given the dependence of post-infarct VTs on poorly-coupled surviving fibres -, and the length ($\sim24-30mm$) and width ($\sim8-11 mm$) of the channels were chosen according to a clinical study(15). The isthmuses of the infarcts were prescribed a CV of $0.30 m/s$ to be able to simulate stable VT episodes with physiological cycles (ranging between $200- 450 ms$ across the five torsos). With the eikonal model, two re-entrant circuits were simulated per scar (with opposing chiralities), as follows: one mouth of the virtual isthmus was temporarily blocked (creating an artificial conduction block orthogonal to the isthmus) **Figure S2A**, and the region of healthy tissue after the block, outside the isthmus, was paced **Figure S2B**, forcing the electrical wave to propagate around the non-conducting scar regions **Figure S2C**. This returned the characteristic figure-of-eight activation pattern. The same procedure was repeated for the other mouth of the isthmus. After a single re-entrant circuit, the tissue set temporarily to be non-conducting at the isthmus mouth was restored to conducting tissue, and diffusion was utilised to maintain the re-entrant circuit for multiple periods (~ $4.6s$) – diffusion coefficients were adjusted so that overall CVs and VT periods were not altered. Finally, ECG and EGM signals were computed as described above. The site of origin (SoO) of each episode – utilised as testing *ground truth* in our DL architecture - was defined as a 5mm-in-radius region around the exit site of the figure-of-eight circuit, to replicate the tip of conventional ablation catheters(16), and hence the minimum ablation lesion size feasible in clinical practice.


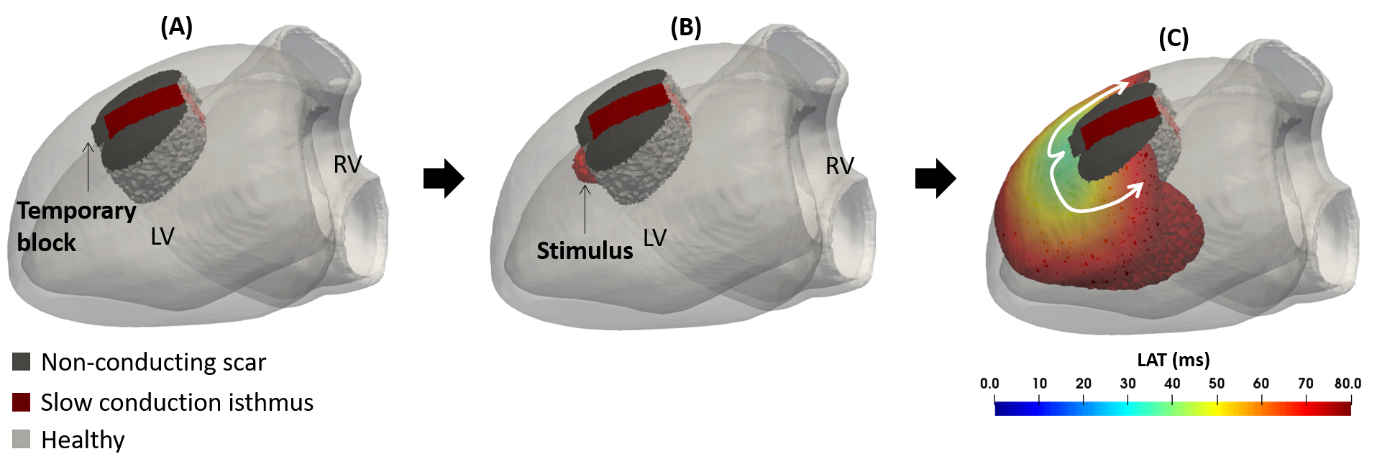


Figure S2. (A): artificial, temporary conduction block; (B): stimulation point; (C) impulse bifurcation. LAT: local activation times.

## **DL Model**

The proposed DL model was designed with reference to (17), with modifications made to fit the present task (**Figure S 3**). Firstly, the model is comprised of two (as opposed to one^13^) identical 1D 8-layer (as opposed to 13-layer^13^) convolutional neural networks (CNN), followed by two long-short term memory (LSTM) cells and an attention mechanism. One of the two architectures classifies the UVC $\varphi$ (discretised into 17 classes) and the other returns UVC $z$ and $\rho$ after a linear regression layer. Differences with (17) are also present in the kernel size and stride of pooling layers (2x2 instead of 3x3), in the absence of batch normalisation, in the number of input cells in LSTMs (128 and 64, instead of 512 and 512), and in the kernel size of the first two convolutional layers ( 5x5 instead of 3x3). Activation functions in each layer (ReLU for CNNs, sigmoid for LSTMs) were not modified, the networks were trained utilising Adam optimiser, and 0.2 dropout was applied in LSTM cells. Learning rates of 0.0001 and 0.0002, and batch sizes of 75 and 100 were utilised for $\varphi$ and $(z,\rho)$ architectures, respectively. Number of epochs were 70 and 50 for $\varphi$ and $(z, \rho)$ networks respectively, but early stopping was prescribed in both. Our 8-layer CNNs follow the well-established 11-layer VGGNET structure(18); 2D convolutions were replaced with 1D, the last four convolutions had a width of 256 instead of 512, the last two pooling layers had a stride of 1, and the last three fully connected layers were replaced with LSTMs and attention, as described above. The DL algorithm was implemented in Python using TensorFlow(19), and the attention layer was exported from (20).


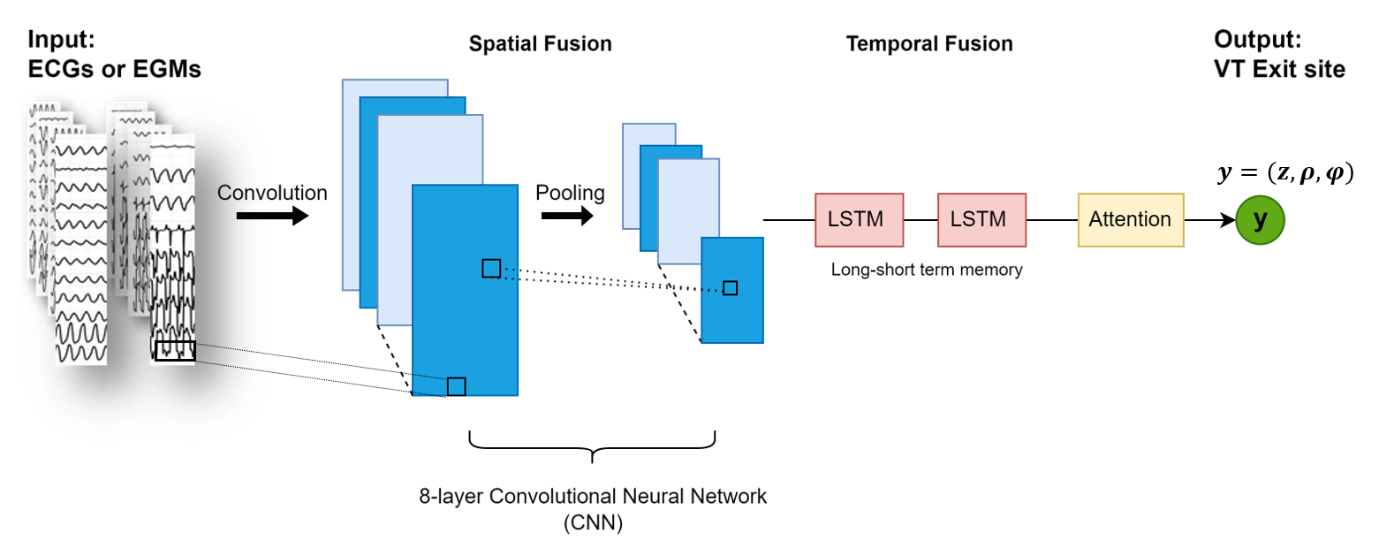


Figure S3. The DL model consists of a 8-layer convolutional neural network (CNN), that allows spatial fusion of the features present in the 16-lead ECGs and/or 8-vector EGMs, followed by two long-short-term memory (LSTMs) cells – allowing temporal analysis of the time traces – and an attention mechanism. The model takes either ECGs or EGMs as inputs, and it predicts the VT exit site in universal ventricular coordinates.

## **Transfer learning**

In this study, we applied transfer learning so we could limit the number of simulations of post-infarct VTs to a total of 500 (which are not as computationally efficient to simulate as focal paced beats), and still achieve an acceptable performance. We re-trained the last 4 convolutional layers of the $\varphi$ and $(z, \rho)$ architectures , with no change in the overall structure and parameters.

## **Data augmentation for training**

Simulated ECGs and EGM traces of the focal paced beats and post-infarct VTs were augmented to increase the training datasets, and robustness of the DL networks. Specifically, QRS complexes of the second paced beats were extracted, and replicated six times to create a total signal of length $2.4s$ including 6 separate QRS complexes. Then, three different $1.5s$ long windows of the signals were taken into consideration (at $t = 0, 240, 690 ms$), to reduce the sensitivity of the algorithms to the starting point of the input signals. In addition, we considered five different levels of white Gaussian noise - signal-to-noise ratio (SNR) of $10, 15, 20, 25, 30 db$. A total of $266,724$ 16-lead ECGs and 8-vector EGMs of simulated focal paced beats were therefore available for initial training of the DL architecture.

For the post-infarct VTs, $4.6s$ long signals (VT periods mentioned above) were stretched/compressed by factors of 0$.9, 1.5 and 1.7$ to increase VT cycles per scar, and added seven different white Gaussian noise SNRs ($5,10, 15, 20, 25, 30,35 db$ ). Seven different $1.5s$ long windows were extracted at $t=0,500, 700, 900, 1100,1300,1500 ms$, as mentioned above. A total of $96,000$ 16-lead ECGs and 8-vector EGMs were available for transfer learning and testing.

## **Testing of simulated post-infarct VTs**

In *Scenario 1*, the DL model was trained on focal paced beats of all five torsos, and then it was re-trained five separate times on post-infarct VTs of four torsos (excluding a different torso every time). These five re-trained models were each tested to localise simulated post-infarct VTs from ECGs/EGMs of the torso excluded during transfer learning. In *Scenario 2,* the DL model was trained five separate times on focal paced beats of four torsos (excluding a different torso every time), and each trained model was re-trained on the post-infarct VTs of the same torsos, and tested on *unseen* post-infarct VTs of the *unseen* torso model. In each testing case, $1.5s$ long 16-lead ECGs and 8-vector EGMs (no noise) of 100 simulated post-infarct VTs were utilised. The DL model was tested at different starting points of the signals, and the predictions of each windows were averaged across the different windows for each torso, and scenario, returning 10 final localisation errors (LEs).

## **References**

1. Monaci S, Strocchi M, Rodero C, Gillette K, Whitaker J, Rajani R, et al. In-silico pace-mapping using a detailed whole torso model and implanted electronic device electrograms for more efficient ablation planning. Comput Biol Med. 2020;125:104005.

2. Monaci S, Gillette K, Puyol-Antón E, Rajani R, Plank G, King A, et al. Automated Localization of Focal Ventricular Tachycardia From Simulated Implanted Device Electrograms: A Combined Physics–AI Approach. Front Physiol. 2021;12:943.

3. Swerdlow CD, Asirvatham SJ, Ellenbogen KA, Friedman PA. Troubleshooting Implanted Cardioverter Defibrillator Sensing Problems I. Circ Arrhythmia Electrophysiol [Internet]. 2014 Dec;7(6):1237–61. Available from: https://www.ahajournals.org/doi/10.1161/CIRCEP.114.002344

4. ACUITY^TM^ X4 Quadripolar LV Leads - Boston Scientific [Internet]. Available from: https://www.bostonscientific.com/en-US/products/leads/acuity-x4-quadripolar-lv-lead.html

5. Plancke AM, Connolly A, Gemmell PM, Neic A, McSpadden LC, Whitaker J, et al. Generation of a cohort of whole-torso cardiac models for assessing the utility of a novel computed shock vector efficiency metric for ICD optimisation. Comput Biol Med. 2019 Sep 1;112:103368.

6. Costa CM, Hoetzl E, Rocha BM, Prassl AJ, Plank G. Automatic Parameterization Strategy for Cardiac Electrophysiology Simulations. Comput Cardiol (2010). 2013;40:373–6.

7. ten Tusscher KHWJ, Noble D, Noble PJ, Panfilov A V. A model for human ventricular tissue. Am J Physiol Circ Physiol. 2004;286(4):H1573–89.

8. Neic A, Campos FO, Prassl AJ, Niederer SA, Bishop MJ, Vigmond EJ, et al. Efficient computation of electrograms and ECGs in human whole heart simulations using a reaction-eikonal model. J Comput Phys. 2017;346:191–211.

9. Potse M. Scalable and Accurate ECG Simulation for Reaction-Diffusion Models of the Human Heart. Front Physiol. 2018 Apr 20;9(APR):370.

10. Gillette K, Gsell MAF, Prassl AJ, Karabelas E, Reiter U, Reiter G, et al. A Framework for the generation of digital twins of cardiac electrophysiology from clinical 12-leads ECGs. Med Image Anal. 2021;71:102080.

11. Bishop MJ, Plank G. Representing cardiac bidomain bath-loading effects by an augmented monodomain approach: Application to complex ventricular models. IEEE Trans Biomed Eng. 2011;58(4):1066–75.

12. Bishop MJ, Plank G. Bidomain ECG simulations using an augmented monodomain model for the cardiac source. IEEE Trans Biomed Eng. 2011;58(8):2297–307.

13. Selvadurai BSN, Puntmann VO, Bluemke DA, Ferrari VA, Friedrich MG, Kramer CM, et al. Definition of Left Ventricular Segments for Cardiac Magnetic Resonance Imaging. JACC Cardiovasc Imaging. 2018;11(6):926–8.

14. Martin R, Hocini M, Haïsaguerre M, Jaïs P, Sacher F. Ventricular Tachycardia Isthmus Characteristics: Insights from High-density Mapping. Arrhythmia Electrophysiol Rev [Internet]. 2019 Mar 12 [cited 2022 Mar 15];8(1):54–9. Available from: https://www.aerjournal.com/articles/ventricular-tachycardia-isthmus

15. De Chillou C, Lacroix D, Klug D, Magnin-Poull I, Marquié C, Messier M, et al. Isthmus Characteristics of Reentrant Ventricular Tachycardia After Myocardial Infarction. Circulation. 2002;105(6):726–31.

16. Ilg K, Baman TS, Gupta SK, Swanson S, Good E, Chugh A, et al. Assessment of Radiofrequency Ablation Lesions by CMR Imaging After Ablation of Idiopathic Ventricular Arrhythmias. JACC Cardiovasc Imaging. 2010 Mar;3(3):278–85.

17. Yao Q, Wang R, Fan X, Liu J, Li Y. Multi-class Arrhythmia detection from 12-lead varied-length ECG using Attention-based Time-Incremental Convolutional Neural Network. Inf Fusion. 2020;53(June 2019):174–82.

18. Simonyan K, Zisserman A. Very Deep Convolutional Networks for Large-Scale Image Recognition. 3rd Int Conf Learn Represent ICLR 2015 - Conf Track Proc. 2014;

19. Abadi M, Agarwal A, Barham P, Brevdo E, Chen Z, Citro C, et al. TensorFlow: Large-scale machine learning on heterogeneous systems [Internet]. 2015. Available from: tensorflow.org

20. Winata GI, Kampman OP, Fung P. Attention-Based LSTM for Psychological Stress Detection from Spoken Language Using Distant Supervision. ICASSP, IEEE Int Conf Acoust Speech Signal Process - Proc. 2018;2018-April:6204–8.
